# Over-expression of RALYL suppresses the progression of ovarian clear cell carcinoma through inhibiting MAPK and CDH1 signaling pathways

**DOI:** 10.7150/ijms.51488

**Published:** 2021-01-01

**Authors:** Ye Xia, Shanting Ye, Yang Yang, Yuchen Liu, Guoqing Tong

**Affiliations:** 1 Reproductive Medicine Center, Shuguang Hospital Affiliated to Shanghai University of Traditional Chinese Medicine, Shanghai 201203, China.; 2 Department of Hepatobiliary Surgery, Shenzhen Second People's Hospital, Clinical Institute of Guangzhou Medical University, Shenzhen 518035, Guangdong Province, China.

**Keywords:** RAYLY, ovarian clear cell carcinoma, MAPK, CDH1

## Abstract

**Background:** The molecular mechanism in the progression of ovarian clear cell carcinoma (OCCC) remains unclear.

**Objective:** This study aimed to investigate the potential function of RAYLY in OCCC.

**Methods:** To validate RAYLY expression, immunohistochemistry, quantitative real-time PCR and western blotting were performed in OCCC tissues and the cell lines of OCCC and epithelial ovarian carcinoma (EOC). Subsequently, the biological effects of RALYL were evaluated through colony formation, and cell proliferation, migration and invasion assays. Finally, RNA-sequencing and gene set enrichment analysis (GSEA) were conducted to explore potential mechanism of RALYL in OCCC.

**Results:** In our study, RALYL was significantly down-regulated in a majority of OCCC tissues compared to adjacent non-tumorous tissues, and OCCC cells had a lower expression level of RALYL than that of EOC cells. OCCC patients with high RALYL expression had a better pathological stage and prognosis. *In vitro*, over-expression of RALYL inhibited cell proliferation, migration and invasion in OCCC. GSEA analysis and western blot indicated an enrichment of MAPK and CDH1 signaling pathways in OCCC cells without RALYL over-expression.

**Conclusions:** RALYL played an important role in the progression of OCCC, and might serve as a potential prognostic biomarker and novel therapeutic target for OCCC.

## Introduction

Epithelial ovarian carcinoma (EOC) is the second most common gynecologic cancer and the leading cause of death for patients with gynecologic cancer in developed countries 1. EOC has four major pathological types including serous, clear cell, endometrioid and mucinous. Of the four subtypes, ovarian clear cell carcinoma (OCCC) is the second most common, and characterized by resistance to conventional platinum-based chemotherapy 2, 3. For recurrent and chemo-resistant OCCC, treatment options are limited to coupling cytotoxic drugs like docetaxel, paclitaxel, and irinotecan with platinum-based therapy 4, 5. Several clinical trials indicated no improvement of these cytotoxic drug cocktails in the OCCC prognosis 6, 7. This underlines the urgent need for better and more effective therapeutic alternatives for OCCC.

As the same cellular subtype in pathology, OCCC has a similar gene expression pattern with renal clear cell carcinoma (RCCC) 8. The effective drug of sunitinib in treating RCCC also demonstrates a definite efficacy in the therapy of OCCC 9-11. In our previous study, we found a significant decrease of RALYL in RCCC for the first time 12. RALYL expression negatively correlated with a poor RCCC prognosis, indicating a potential therapeutic target of RALYL in clear cell carcinoma. However, the anti-tumor mechanism remains unclear, as well as its role in OCCC. In this study, we investigated the aberrant expression of RALYL in OCCC and its correlation with clinical characteristics. Additionally, we also performed a series of *in vitro* experiments to elucidate potential biological function of RALYL in OCCC.

## Materials and methods

### Tissue sample collection

Tumor and matched adjacent non-tumor tissue samples of 40 OCCC patients were collected from Department of Gynaecology, Shuguang Hospital (Shanghai, China) between April 2010 and September 2019, and immediately stored in liquid nitrogen. All samples were obtained with informed consent, and our study was approved by the ethic committee of Shuguang Hospital, Shanghai University of Traditional Chinese Medicine.

### Immunohistochemistry (IHC)

Paraformaldehyde-fixed, paraffin-embedded and sectioned samples were blocked in 5% BSA for 40 min, included with the primary antibody against human RALYL (1:100, Abcam) overnight at 4 °C, and sequentially incubated with CY3-conjugated anti-rabbit secondary antibody (Servicebio, China) for 1 h at room temperature. IHC images were obtained under the microscope (Olympus, Japan), and Image-Pro Plus 6.0 software (Media Cybernetics, USA) was used to quantify the corresponding staining.

### Cell culture and transfection

The OCCC cell line ES-2 and the EOC cell line A2780 were purchased from the Cell Resource Center of Shanghai Institutes for Biological Sciences (Shanghai, China). Cells were cultured in DMEM (HyClone, USA) containing 10% FBS (Gibco, USA) and 1% penicillin-streptomycin (HyClone, USA) at 37 °C in a 5% CO_2_ atmosphere. Full-length RALYL cDNA (915bp) was inserted into pLVX-IRES-NEO vector (pLVX-IRES-NEO-RALYL) (Clontech, USA). Lentiviral vectors packaged with RALYL and negative control vectors were respectively transfected to cells using Lipofectamine 2000 Reagent (Invitrogen, USA).

### Quantitative real-time PCR

Total RNA was extracted from tumor tissues and cells using TRIzol reagent (Invitrogen, USA), and then reversely transcribed to cDNA by ReverTra Kit (TOYOBO, Japan). Quantitative real-time PCR was conducted with SYBR Premix Ex Taq Kit (TaKaRa, Japan) in Applied Biosystems 7300 System (Thermo, USA). The expression levels were calculated using the comparative CT method for relative quantification against GAPDH.

### Western blotting

Total protein in tumor tissues and cells was extracted using RIPA lysis buffer (Beyotime, China). Protein lysates were separated by SDS-PAGE, transferred to PVDF membranes (Millipore, USA), and blocked with 5% BSA (Biofroxx, Germany). The membranes were incubated with the primary antibodies overnight at 4 °C, and subsequently with the anti-rabbit HRP-conjugated secondary antibody (Servicebio, China). Chemiluminescent signals were detected by Enhanced Chemiluminescence (Thermo, USA).

### Colony formation assay

Cells in the logarithmic growth phase were seeded into a 6-well plate at 1000 cells/well, and cultured at 37 °C with 5% CO_2_ for 2 weeks. Then, the cells were fixed with 4% paraformaldehyde and stained with crystal violet. Colonies with at least 50 cells were counted under the microscope.

### Cell proliferation assay

Cell proliferation was detected using CCK8 assay (Sigma, USA). Cells were seeded in a 96-well plate at 5000 cells/well, and incubated at 37 °C for 1, 2, 3, 4 and 5 days respectively. At each time point, the cells were incubated with 0.5% MTT for 4 h, and then lysed with DMSO (Sigma, USA). The absorbance value (OD) at 490 nm was measured using a microplate reader (BioTek, USA).

### Cell migration and invasion assay

Cell migration and invasion were determined using Transwell assay (Corning, USA). Cells were diluted with serum-free medium to 1×10^5^/ml. 500 μl of the suspension were transferred to the upper chamber, and 750 μl medium containing 10% FBS was added into the lower chamber. Transwell plate was incubated at 37 °C for 24 h, fixed with 4% paraformaldehyde for 30 min and stained with 5% crystal violet for 30 min. Cells were counted under the microscope for quantification.

### RNA-sequencing and GSEA analysis

Illumina HiSeq 4000 (Illumina, USA) was used to perform paired-end transcriptome sequencing in the samples of ES-2 cells with and without RALYL transfection. In quality control, the reads with adapter contamination, undetermined bases and low-quality bases were removed by the Cutadapt software (Martin, 2011). The remaining reads were aligned to the human genome sequences (GRCh38.p12) using the HISAT software (Johns Hopkins University, USA). The expression levels of all transcripts were determined by calculating the FPKM.

To identify potential function of RALYL, gene set enrichment analysis (GSEA) was conducted to detect biological processes enriched in the samples with RALYL highly expressed. False discovery rate (FDR) <0.05 was chosen as the cut-off criteria.

### Statistical analysis

All data were presented as mean ± SD unless otherwise indicated. The statistical difference between groups was assessed by unpaired two-tailed Student's *t* test using GraphPad 6.0 software (GraphPad, USA). Two-tailed *P* values < 0.05 were considered statistically significant. Differences among three or more groups were assessed by one-way ANOVA test. Pearson's *χ^2^* test was used to evaluate the correlation of clinical characteristics between two groups with differential expression. Survival analysis was carried out using the KM method, and the log-rank method was used to identify the difference in survival curve. *P* values < 0.05 were considered statistically significant.

## Results

### RALYL is down-regulated in OCCC

**Figure 1:**
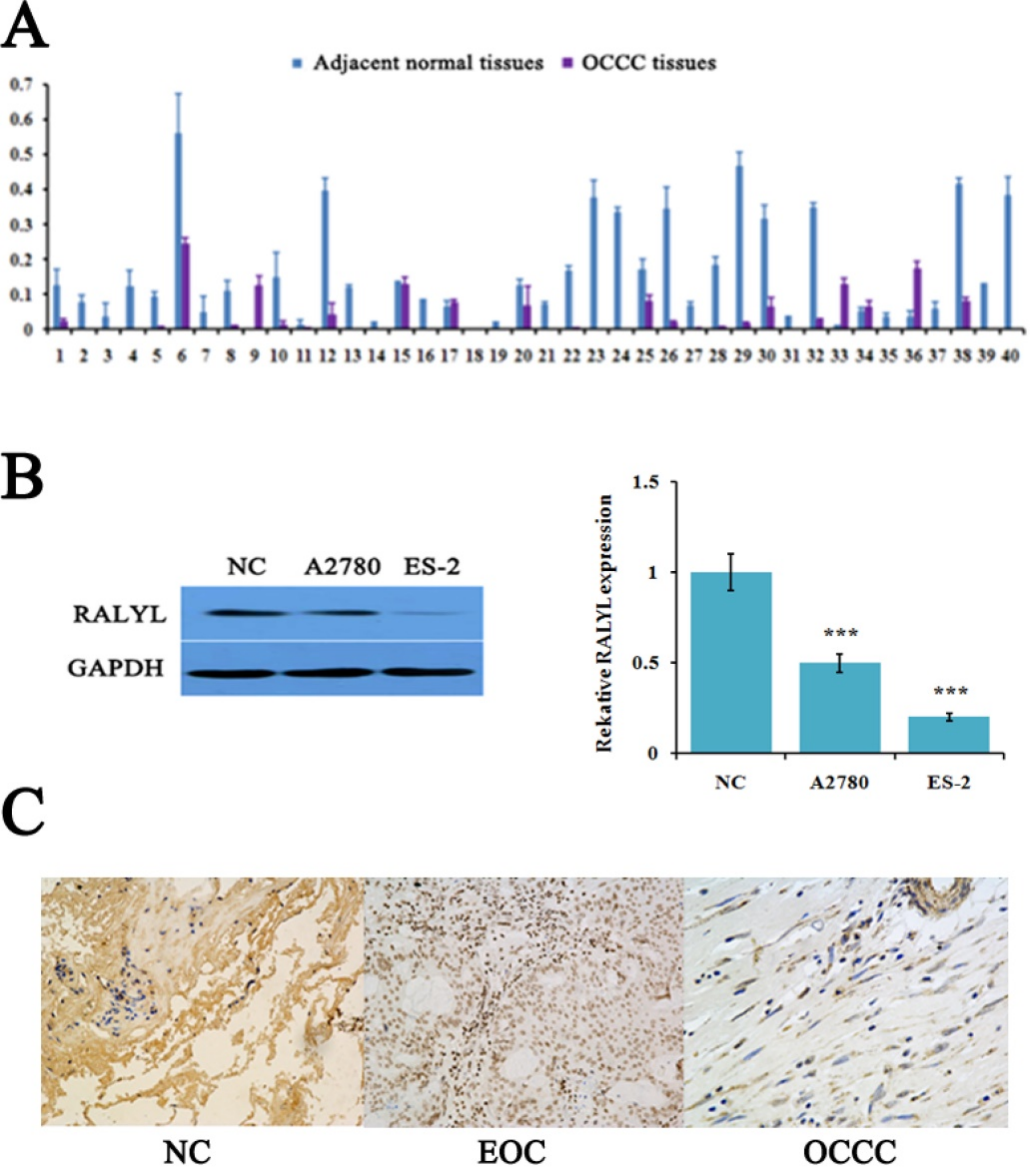
** The** expression of RALYL in ovarian clear cell carcinoma (OCCC). (A) Relative expression of RALYL in 40 pairs of OCCC tissues. (B) Relative expression of RALYL in the OCCC cell line ES-2 and the epithelial ovarian cancer (EOC) cell line A2780. (C) Immunohistochemistry analysis of RALYL in OCCC tissues. *P<0.05, **P<0.01, ***P<0.001.

The expression level of RALYL detected by quantitative real-time PCR showed that RALYL was significantly down-regulated in a majority of OCCC tissues compared to adjacent non-tumorous tissues (**Figure 1A**). Subsequently, we evaluated RALYL expression in the OCCC cell line ES-2 and the EOC cell line A2780. As shown in **Figure 1B**, RALYL expression was obvious down-regulated in both ovarian cancer cell lines when compared to the normal ovarian cell line. Furthermore, ES-2 cells had a lower expression level of RALYL than that of A2780. In OCCC tissues, IHC analysis indicated RALYL protein was located in the cell membrane and cytoplasm (**Figure 1C**).

### RALYL correlates with the clinical characteristics of OCCC

**Figure 2:**
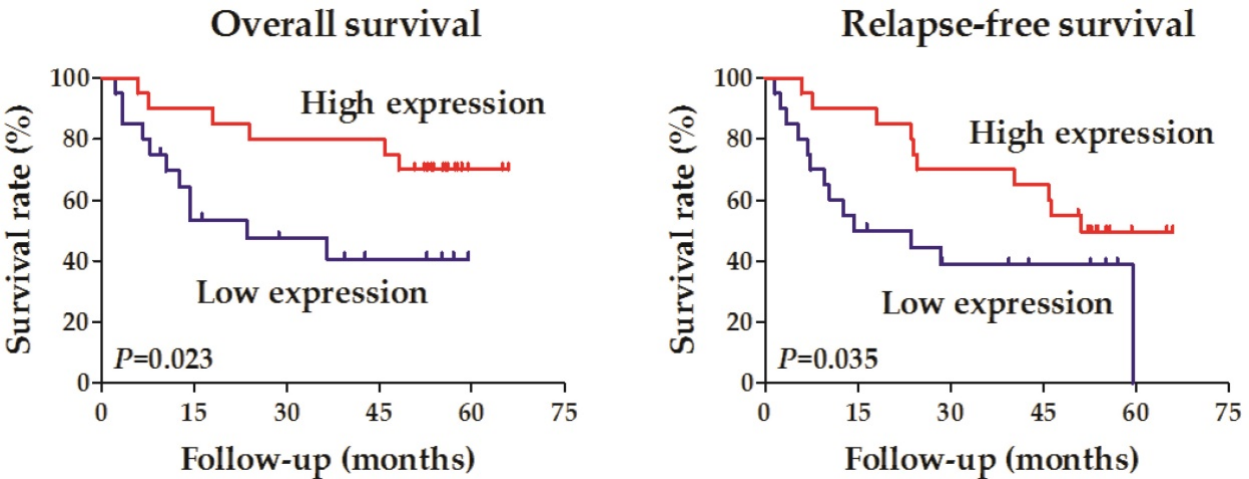
Survival analysis of the association between RALYL expression and the prognosis in ovarian clear cell carcinoma.

Forty OCCC samples were divided into two groups according to the expression level of RALYL (high *vs.* low expression). Correlation analysis was conducted to evaluate the relationship between RALYL expression and the clinical characteristics of OCCC, including age, pathological differentiation, tumor depth, lymph node metastasis, distant metastasis, and tumor stage (**Table 1**). The results showed that RALYL expression had a strong negative correlation with the tumor depth and stage (*P*<0.05). Survival analysis also indicated a better prognosis in the RALYL high expression group, including both overall survival and relapse-free survival (**Figure 2**).

### RALYL inhibits OCCC cell proliferation

**Figure 3:**
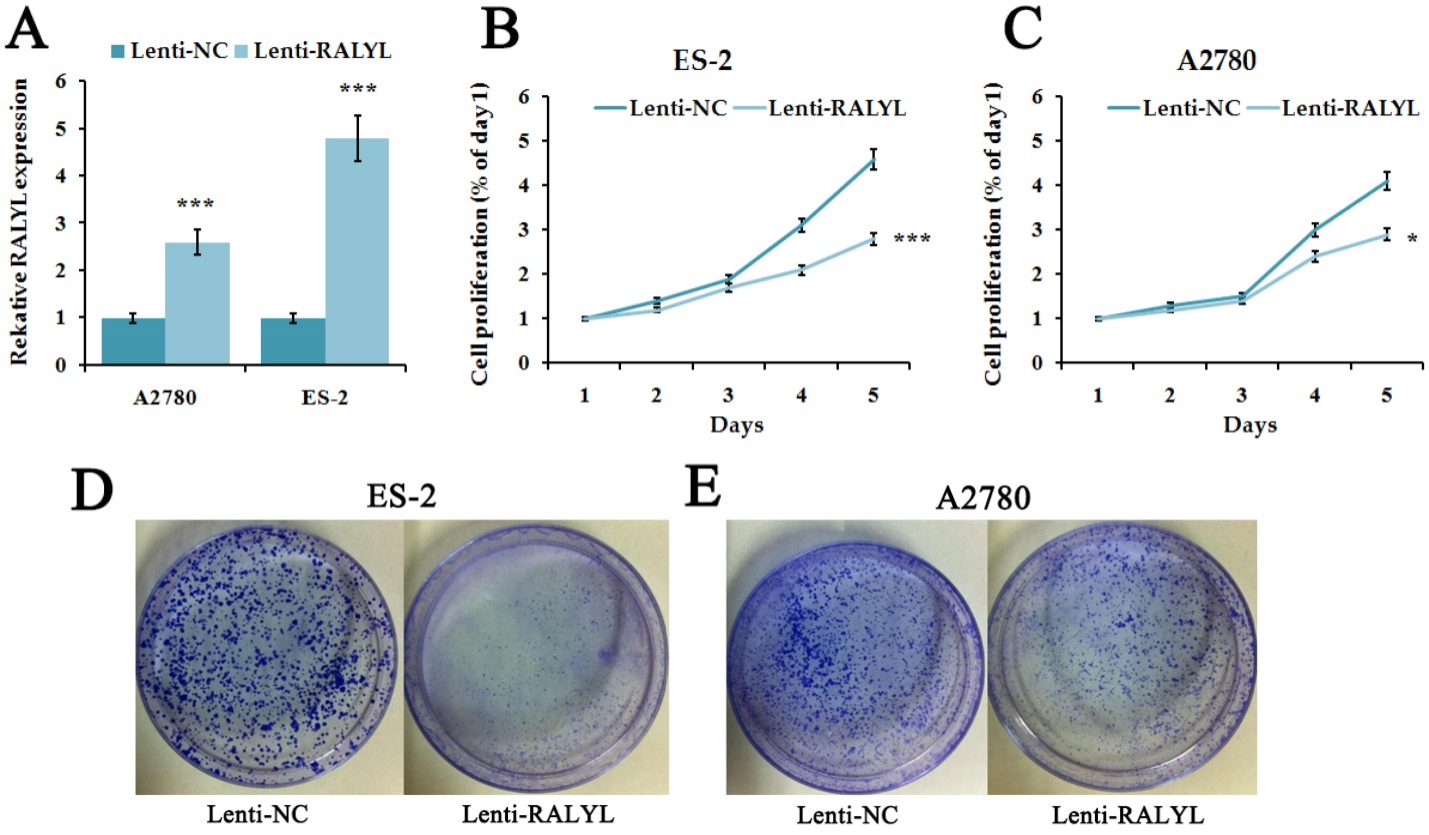
The effects of RALYL over-expression on OCCC cell proliferation. (A) The transfection efficiency of lentiviral vectors packaged with RALYL in ES-2 and A2780. (B-C) CCK-8 assay analysis of lenti-RALYL transfected ES-2 cells and A2780 cells. (D-E) Colony formation assay analysis of lenti-RALYL transfected ES-2 cells and A2780 cells. *P<0.05, **P<0.01, ***P<0.001.

Since RALYL was down-regulated in OCCC, we further investigated its biological function. We over-expressed RALYL in OCCC cells by transfecting lentiviral vectors packaged with RALYL. Quantitative real-time PCR and western blot were conducted to verify the over-expression efficiency of RALYL (**Figure 3A**). To detect whether RALYL regulated the proliferation of OCCC cells, we performed CCK8 and colony formation assay. The results showed that the growth capacity and colony number of ES-2 cells and A2780 cells dramatically decreased following over-expression of RALYL (**Figure 3B-3E**). Compared to A2780 cells, the decrease was more significant in ES-2 cells after over-expression of RALYL.

### RALYL inhibits the migration and invasion of OCCC cells

**Figure 4:**
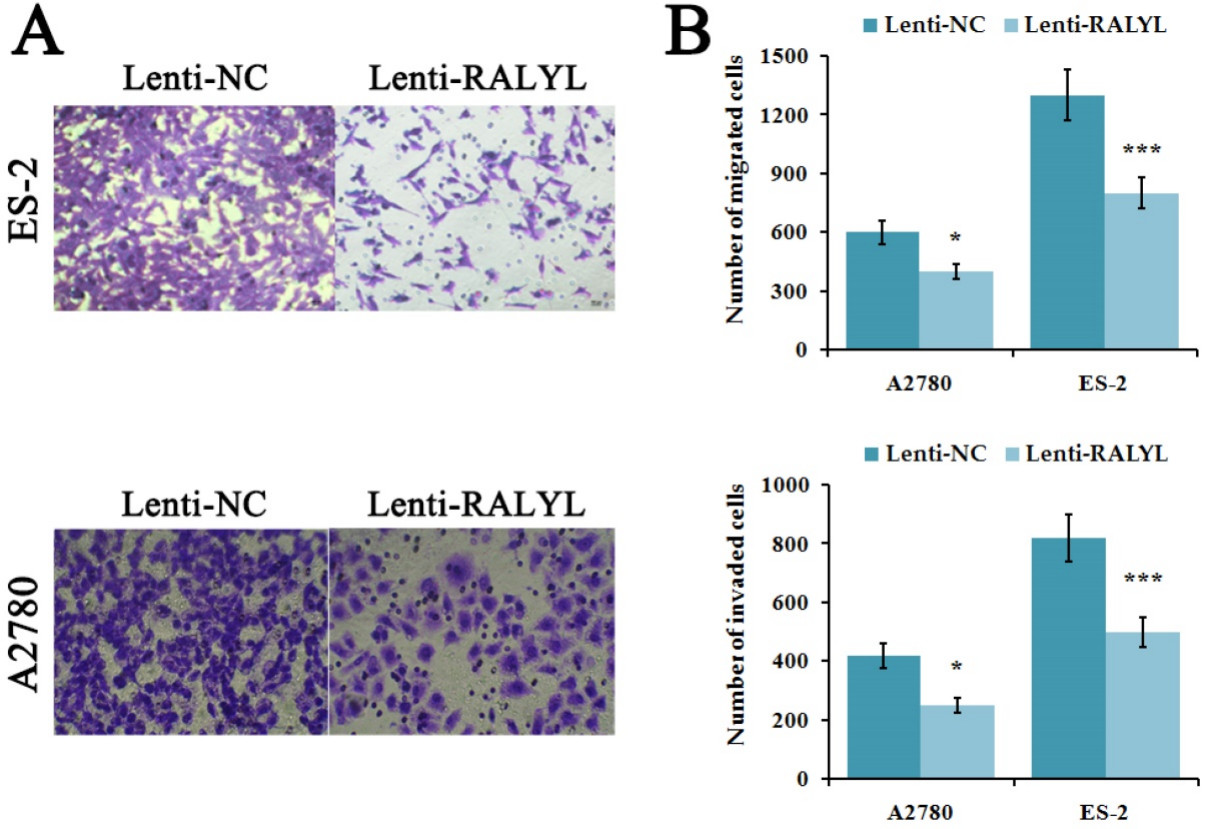
Effects of RALYL over-expression on the migration and invasion of OCCC cells. (A) Migration and invasion assay analysis of lenti-RALYL transfected ES-2 cells and A2780 cells. (B) Migrated and invaded cells transfected with lenti-RALYL were counted. *P<0.05, **P<0.01, ***P<0.001.

To evaluate the effects of RALYL on metastasis ability of OCCC cells, cell migration and invasion assay were performed. The results indicated a significant decrease of migrated and invaded cells in ES-2 cells and A2780 cells after over-expression of RALYL (**Figure 4A-4B**). Compared to A2780 cells, the suppression was more significant in ES-2 cells after over-expression of RALYL.

### RALYL inhibits MAPK and CDH1 signaling pathways in OCCC

**Figure 5:**
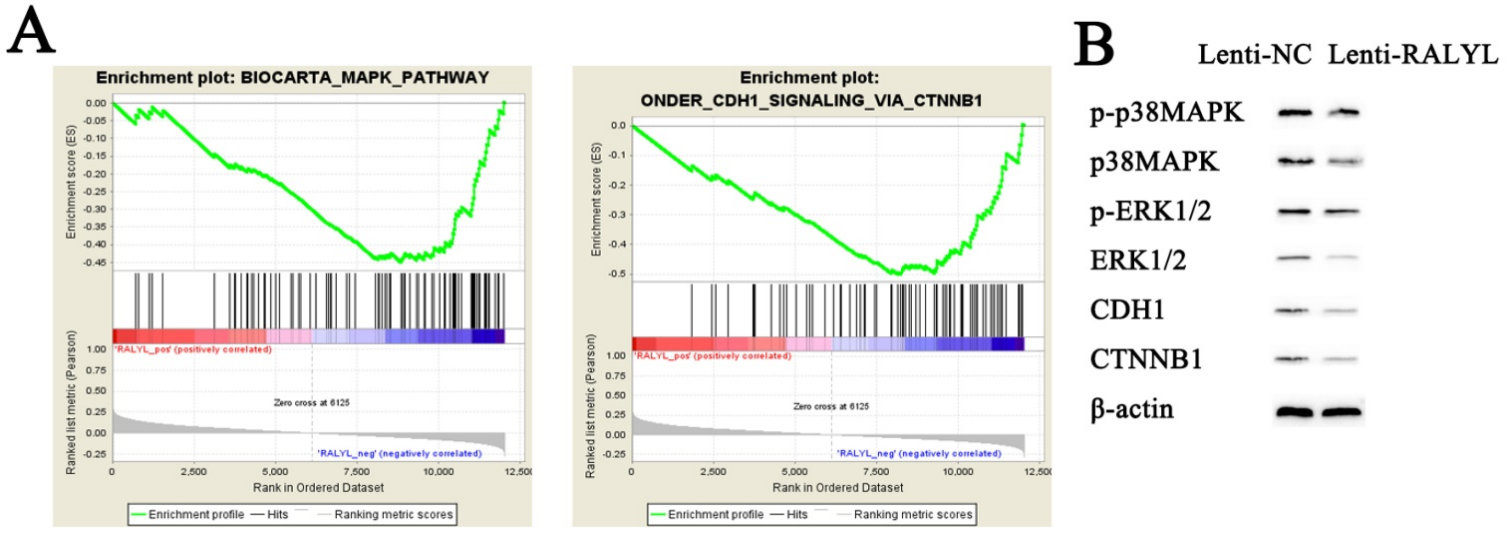
Inhibition of MAPK and CDH1 signaling pathways by RALYL over-expression in OCCC. (A) Gene set enrichment of biological processes associated with RALYL. (B) The effects of RALYL over-expression on MAPK and CDH1 signaling pathways.

To identify potential function of RALYL in OCCC, we conducted RNA-sequencing of ES-2 cells treated with the RALYL over-expression vector and the control vector. The standardized expression data were used to perform GSEA analysis, which could help detect biological processes enriched in the samples with RALYL over-expression. The results demonstrated an enrichment of MAPK and CDH1 signaling pathways in ES-2 cells after over-expression of RALYL (**Figure 5A**). Collectively, these findings implied an involvement of RALYL in the OCCC pathogenesis by targeting MAPK and CDH1 signaling pathways, which have been reported to play a key role in the tumorigenesis (13, 14). To verify our speculation, western blot was conducted to investigate the expression levels of proteins in the signaling pathways. The results indicated a significant decrease of p-p38MAPK, p38MAPK, p-ERK1/2, ERK1/2, CDH1 and CTNNB1 in ES-2 cells after over-expression of RALYL (**Figure 5B**).

## Discussion

In this study, we found an obvious down-regulation of RALYL in OCCC tissues, and the OCCC cell line also showed a more significant decrease in RALYL expression than that of the EOC cell line. In clinical analysis, RALYL expression had a strong negative correlation with the tumor depth and stage, and the patients with high RALYL expression demonstrated a better prognosis. These findings indicated an anti-tumor role of RALYL in OCCC. To validate its biological function, CCK8, colony formation assay, cell migration and invasion assay were conducted, and we found a significant inhibition in the OCCC cells after over-expression of RALYL. Furthermore, compared to the EOC cells, the suppression was more significant in OCCC cells after over-expression of RALYL. To investigate the mechanism, we performed a RNA-sequencing and GSEA analysis, and found an enrichment of MAPK and CDH1 signaling pathways in the OCCC cells overexpressing RALYL. Generally, RALYL played an anti-tumor role in OCCC, and might sever as a therapeutic and prognostic biomarker for OCCC.

RALY RNA binding protein-like (RALYL) belonged to the RALY subfamily, and it contained ten exons and one RRM (RNA recognition motif) domain so as to combine the RNA. The microarray expression date showed that RALYL were highly expressed in the normal adrenal, kidney and brain, and low RALYL expression was correlated with mental disorder, Parkinson's disease, and multiple cancers 15-17.

After searching protein-protein interaction database of STRING (https://string-db.org/), we found that RALYL could interact with multiple proteins, like TIGD3, RALY, RBM22, HNRNPCL1, HNRNPCL2, HNRNPC, CRNKL1, OR52N5, PNN and PPIE. RALY was up-regulated in multiple cancers, and RALY over-expression led to an aggressive biological behavior and a dismal prognosis as well as activating CDH1 signaling pathway 18-20. By specific binding with RALY, RALYL could inhibit the oncogenic effects of RALY. Furthermore, HNRNPC, CRNKL1, PNN and PPIE were also reported to have a high expression and biological function in several cancers including ovarian cancer 21-24. RALYL might also play the anti-tumor effects by suppressing these oncogenic genes.

In conclusion, our study revealed thar RALYL was down-regulated in OCCC, and patients with RALYL low expression had a poor pathological stage and prognosis. In addition, over-expression of RALYL showed an obvious anti-tumor effect by inhibiting MAPK and CDH1 signaling pathways.

## Figures and Tables

**Table 1:** Correlation between RALYL expression and clinicopathological characteristics in patients with ovarian clear cell carcinoma

Characteristics	Number (n=40)	Percent	RALYL		*P* value
			High	Low	
**Ages (years)**					
<60	11	27.5%	6	5	0.724
>60	29	62.5%	14	15	
**Pathological differentiation**					
Well and moderate	17	42.5%	9	8	0.749
Poor	23	57.5%	11	12	
**Tumor depth**					
T1 and T2	19	47.5%	13	6	0.027
T3 and T4	21	52.5%	7	14	
**Lymph node metastasis**					
N0	25	62.5%	14	11	0.327
N1	15	37.5%	6	9	
**Distant metastasis**					
M0	39	97.5%	20	19	0.311
M1	1	2.5%	0	1	
**Tumor stage**					
I and II	22	55.0%	13	9	0.027
III and IV	18	45.0%	7	11	
